# One-Year Outcome of Geriatric Hip-Fracture Patients following Prolonged ICU Treatment

**DOI:** 10.1155/2016/8431213

**Published:** 2016-01-13

**Authors:** Daphne Eschbach, Christopher Bliemel, Ludwig Oberkircher, Rene Aigner, Juliana Hack, Benjamin Bockmann, Steffen Ruchholtz, Benjamin Buecking

**Affiliations:** Center for Orthopaedics and Trauma Surgery, University Hospital Giessen and Marburg GmbH, Location Marburg, Baldingerstraße, 35043 Marburg, Germany

## Abstract

*Purpose*. Incidence of geriatric fractures is increasing. Knowledge of outcome data for hip-fracture patients undergoing intensive-care unit (ICU) treatment, including invasive ventilatory management (IVM) and hemodiafiltration (CVVHDF), is sparse.* Methods*. Single-center prospective observational study including 402 geriatric hip-fracture patients. Age, gender, the American Society of Anesthesiologists (ASA) classification, and the Barthel index (BI) were documented. Underlying reasons for prolonged ICU stay were registered, as well as assessed procedures like IVM and CVVHDF. Outcome parameters were in-hospital, 6-month, and 1-year mortality and need for nursing care.* Results*. 15% were treated > 3 days and 68% < 3 days in ICU. Both cohorts had similar ASA, BI, and age. In-hospital, 6-month, and 12-month mortality of ICU > 3d cohort were significantly increased (*p* = 0.001). Most frequent indications were cardiocirculatory pathology followed by respiratory failure, renal impairment, and infection. 18% of patients needed CVVHDF and 41% IVM. In these cohorts, 6-month mortality ranged > 80% and 12-month mortality > 90%. 100% needed nursing care after 6 and 12 months.* Conclusions*. ICU treatment > 3 days showed considerable difference in mortality and nursing care needed after 6 and 12 months. Particularly, patients requiring CVVHDF or IVM had disastrous long-term results. Our study may add one further element in complex decision making serving this vulnerable patient cohort.

## 1. Introduction

By 2030, 25% of the western European population is expected to be at least 65 years of age [1]; by 2050, the elderly population will almost triple [2].

Until the age of 85, 11% of women and 5% of men are hospitalized because of femoral fractures [3, 4]. Long-term mortality in this cohort, compared with people of the same age without fracture, is 1.15 : 1, a 20% increase [5]. Thus, successful treatment of these fractures is becoming increasingly important. Over the last years, several models of shared orthogeriatric care have been developed worldwide to improve patients' outcomes. As part of these models, a perioperative observational period was developed as a standard procedure for this patient sample. Monitoring in the postoperative period is also part of current guidelines [6, 7]. Triage studies have demonstrated that patients admitted to ICUs have improved survival compared to rejected patients [8–10]. Elderly patients may have worse prognoses because of more comorbid conditions and fewer physiologic reserves. Some authors recently documented age as an independent risk factor for mortality [11, 12] but not always a factor in predicting worse outcome [13]. Today, plenty of data concerning comorbidities and chronic health conditions identified as risk factors, especially for hip-fracture patients' outcomes, are available. They are associated with poor outcomes (i.e., prolonged hospitalization, higher complication rate, poorer functional levels, and increased mortality) [14, 15]. With the current aging population, more complex procedures, and increasing expectations, demand for ICUs will increase further. Particularly, pulmonary complications and renal failure due to a preexisting chronic failure are severe and life-limiting complications. It is reported that incidence of hospitalization secondary to community-acquired pneumonia doubles in patients aged > 60 years [16]. Posthospitalization outcome data following ICU-dependent complications in geriatric trauma patients are sparsely available. In particular, outcome data after invasive ventilation and filtration due to acute renal failure do not exist for this kind of cohort. Furthermore, short-term survival is probably not the most important factor considered when making treatment decisions in this cohort; we suggest that invasive ICU procedures may increase the likelihood of less favorable post-ICU outcomes such as persistent need of nursing care.

Accordingly, dependent on reasons and assessed procedures for an ICU treatment extending past the normal postoperative period of 1 to 3 days, we aimed to detect considerable differences in 6- and 12-month mortality as well as persistent need of nursing care of these patients.

## 2. Patients and Methods

Patients at least 60 years old with proximal femoral fractures (ICD 10 S 72.0–72.2) were included in this prospective single-center observational study. Research nurses and/or senior physicians collected data.

Criteria for exclusion were multiple traumas (ISS ≥ 16) and malignoma-associated fractures. All patients were surgically treated with either internal fixation or hip arthroplasty. The inclusion period was from April 1, 2009, to September 30, 2011. We obtained the approval of the Ethics Committee of the University of Marburg (AZ 175/08). All of the patients or their legal representatives gave their written consent.

We documented patient age, gender, the American Society of Anesthesiologists (ASA) classification, and fracture type. Patients were requested to give information about their prefracture functional status. We measured the functional status by the Barthel index (BI, Hamburg Classification Manual). We registered the prevalence of ICU stay, underlying reasons for admission, and length of stay in the ICU during the hospitalization period and assessed procedures like invasive ventilator management (IVM) and renal failure demanding CVVHDF. According to further evaluation, we subdivided our patients into 3 groups: those who were not treated in the ICU (nICU, i.e., no intensive-care unit), those who stayed 1 to 3 days in the ICU (sICU, i.e., short intensive-care unit), and those who stayed > 3 days (pICU, i.e., prolonged intensive-care unit). We defined outcome parameters as in-hospital mortality, 6-month mortality and nursing dependency, and 1-year mortality and nursing dependency.

We collected data in a FileMaker database (FileMaker, Inc. 5201 Patrick Henry Drive Santa Clara, CA 95054, USA). We performed double entry with a plausibility check to improve data quality. We used Predictive Analysis Software (PASW) version 22.0 (SPSS Inc., Chicago, IL, USA) for descriptive statistics and explorative data analysis, with the results being presented as numbers and percentages or as means, standard deviations, and 95% confidence intervals. We tested numerous data using the Wilcoxon test or the *t*-test, depending on the Kolmogorov-Smirnov test for normal distribution. We tested all dichotomies using Fisher's exact test. The outcome parameters in hospital mortality, 6-month mortality, and 12-month mortality were analyzed additionally by multivariate analysis addressing the covariates age, gender, BI, ASA, Charlson index, BMI, time until operation, and ICU cohort. For all tests, we assumed statistical significance at *p* ≤ 0.05.

## 3. Results

In the observational period, we were able to include 402 patients. The baseline characteristics of all patients are illustrated in Table 1. Patients underwent operative treatment for hip-related fracture (intramedullary nail/hemiarthroplasty) in the first 24 h. The pICU cohort was operated with a slight delay compared to the sICU cohort (17.2 ± 0.8 h versus 23.3 ± 2.3 h, *p* = 0.041).

We observed 336 (85%) of all patients in the ICU postoperatively for a mean of 2.5 (±3.7) days. The pICU group included 61 patients. None of the patients who were admitted directly to the standard-care unit required ICU admission in the course of inpatient care.

Patients with ICU stays were significantly older than those in group nICU (79 ± 8 versus 82 ± 9, *p* = 0.008). The ICU cohorts showed significantly more female patients and significantly higher ASA scores (*p* ≤ 0.001). Comparing the pICU cohort to the sICU cohort, the differences concerning the abovementioned characteristics seem to be sparse. Both groups had similar ASA, BI, and age. Nevertheless, the pICU cohort showed a significantly higher amount of prefracture nursing care needed, including 144 (52%) in need of care versus 44 (72%) patients in the sICU cohort (*p* = 0.042). CCI was also increased significantly in the pICU cohort (*p* ≤ 0.001).

Comparing the data of further clinical courses, the in-hospital mortality of the pICU cohort was significantly increased (3% versus 26%). The 6- and 12-month mortality were equally increased and statistically significantly compared to the cohort of nICU patients and sICU patients as well (18% versus 48% and 26% versus 59%). The 6-month and 12-month nursing-care need showed equal tendency (67% versus 85% and 66% versus 84%) without reaching a statistically significantly higher amount in the pICU cohort (*p* = 0.072 versus *p* = 0.123; Table 2).

A multivariate analysis for the mortality endpoints “in-hospital mortality,” “6-month mortality,” and “12-month mortality,” including the variables age, gender, BI, ASA, Charlson index, BMI, time until operation, and ICU cohort, was performed. As expected, the ASA and ICU cohorts were independent risk factors affecting the different mortality endpoints. The Charlson index became significant in the 6- and 12-month mortality analysis (Table 3).

As mentioned above, 61 patients were treated for more than 3 days in our ICU. Regarding the underlying reasons concerning a prolonged ICU treatment period, we noticed the main treatment diagnosis during stay: the largest group of 17 patients suffered from cardiac pathology subdivided into arrhythmia, ischemia, or failure. Two patients in this subgroup underwent cardiopulmonary resuscitation (CPR), 1 following ventricular fibrillation and 1 further following myocardial failure with cardiac arrest. The second largest group included primary respiratory failure (*N* = 10), renal impairment (*N* = 8), and infection (*N* = 9) as further numerous events demanding prolonged ICU treatment. Analytic separation of the underlying reasons is given in Table 4.

Following the further clinical course of these 61 patients, 11 (18%) of them received at least transient CVVHDF and 25 (41%) received IVM. In-hospital mortality showed to be 82% in the CVVHDF group and 68% in the IVM group. The 6-month mortality (82% versus 88%) and 12-month mortality (91% versus 92%) ranged in both groups at equal levels without displaying statistical significance. Amounts of 100% are displayed concerning the 6-month and 12-month nursing-care need (Table 5).

## 4. Discussion

With this prospective observational trial, we aimed to investigate long-term outcomes of geriatric hip-fracture patients who had had prolonged treatment in an ICU, particularly with regard to those undergoing invasive ventilation and renal filtration.

There are plenty of studies published that have focused on the meticulous demands of geriatric trauma patients in the last few years. Differentiated outcome data concerning geriatric hip-fracture patients, especially following invasive ventilation and hemodiafiltration due to acute lung failure or renal failure, did not exist for this special kind of cohort.

We know that, following hospital discharge, elderly patients are 2.3 times more likely to die in the long term compared to a similar age group in the general population [17]. A recent study dealing with the long-term outcome of elderly, critically ill patients showed 1-year mortalities after ICU treatment in 76% of unplanned geriatric trauma patients and 46% in planned orthopedic patients [18]. This fits our results of unplanned geriatric trauma patients in the pICU cohort, showing a 1-year mortality rate of 59%.

Most studies do not differentiate between short- and long-term ICU treatment, very likely because the admission diagnoses of most collectives are composed inhomogeneously, displaying the ICU stay itself as a joint feature. Like mentioned above, we subdivided the ICU collective into sICU and pICU cohorts. This decision was made because we saw a great difference in the underlying ICU treatment indications in these 2 groups. Patients in the sICU cohort were often admitted for a short period of postoperative inotropic support or with postoperative bleeding with a need for transfusion due to the intake of blood-thinning medications. Admission diagnoses in the pICU group often required more extensive care, as shown in Table 4.

Plenty of confounders influence the outcome of geriatric hip-fracture patients. Reducing the time between admission and operative treatment is seen as one important factor for improving survival. There are many articles that have assessed this, and a consensus has been agreed upon that a delay of more than 48 hours is unfavorable to survival [19, 20]. Some new studies focusing on 30-day mortality after adjusting for well-known risk factors, such as age, comorbidities, and gender, could show an elevated mortality following more than 12 hours of delay to surgery [21] or even an elevated 90-day mortality following a delay of more than 24 h [22]. As mentioned in Section 3, all patients underwent operative treatment for hip-related fracture in between the first 12–24 h. The pICU cohort was operated on with a slight but significant delay compared to the sICU cohort (17.2 ± 0.8 h versus 23.3 ± 2.3 h, *p* = 0.041). Thus, this significance may influence mortality rates in between our groups, even though this difference of a few hours doubtfully can be considered to display clinical relevance.

As expected, CCI was significantly higher in the pICU cohort, and higher CCI presented as an independent risk factor for higher 6- and 12-month mortality in performed multivariate analysis (Table 3). This higher level of comorbidities is well known to be associated with poorer outcomes in hip-fracture patients [23]. As obese and old patients are known to be more likely to develop adverse outcomes following a primary total-hip replacement, BMI was taken into account as well. Patients of all groups showed rather normal weight or underweight, displaying no statistical significance. The variables age, ASA score, BI, and gender in nICU, sICU, and pICU cohorts showed no statistically significant differences as well.

Apart from all these known confounders for adverse outcomes, we assume that 1 further, huge factor of influence for prolonged ICU treatment was displayed by the demand of ICU-dependent procedures like invasive ventilation or hemodiafiltration.

Particularly, pulmonary complications and renal failure due to a preexisting chronic failure appeared to be severe and life-threatening complications in geriatric trauma patients. Some prospective studies focusing on the influence of age on the outcome of mechanically ventilated elderly patients showed that age has an important effect on the outcome of mechanically ventilated patients [24, 25]. In our collective, in-hospital mortality occurred in 68% of patients with mechanical-ventilation-dependent respiratory complications. This impressively illustrates the fatal consequence of this “third hit,” following trauma as a “first hit” and operative treatment as a “second hit.” Ninety-two percent of these patients died within the first year after admission, and all survivors showed a need for nursing care after 12 months.

Concerning renal failure, recent epidemiological studies have reported an association between reduced glomerular filtration rate and increased risk of death and cardiovascular events [26], as well as the association between renal impairment, frailty, and quality of life. Elderly people with chronic renal insufficiency are known to have a high prevalence of frailty, which may signal their risk for progression to adverse health outcomes [27]. Irrespective of this known data, the finding that only 1 out of 11 patients undergoing hemodiafiltration during ICU stay in our collective survived the first year underlines the severe prognostic character of this procedure.

After all, keeping in mind that only 8% of patients with invasive ventilatory management and 9% of patients undergoing CVVHDF survived the first year—and none of them survived without nursing-care need—prognosis in cases of such complications worsens remarkably. Over the years, plenty of studies have confirmed that the majority of patients do not wish to survive if they lose their independence, if they become a burden on their families, or if they are unable to retain their capacity to think clearly [28, 29]. A current empirical analysis showed that if treatment is invasive and the predicted outcome is survival with severe functional impairment or cognitive impairment, 74.4% and 88.8% of patients surveyed, respectively, would not choose treatment [30]. There is a bright recognition that the burden of the proposed treatment and the probability of adverse outcomes should be specifically discussed with relatives or legal representatives when talking about end-of-life decisions in the course of intensive-care therapy [31]. Particularly, long-term results after invasive ventilation or hemodiafiltration during acute-care treatment were disastrous in our cohort. These data have to be taken into account, since short-term survival is probably not the most important factor considered when making treatment decisions in this vulnerable cohort of geriatric trauma patients. Clinicians, as well as relatives, should be aware that a patient's appraisal about what constitutes an acceptable long-term outcome may change with advancing age.

The present study has some limitations: Cohorts requiring CVVHDF and IVM were small and preexisting medical conditions and admission diagnoses were manifold. Further, because of different healthcare conceptions in different European countries (and even between different provinces in Germany), a comparison of duration of ICU stay and hospitalization period with other studies is not reasonable overall. Finally, due to the high mortality rate after 1 year, the claimed 1-year nursing-care status is certainly emphasizing a worse outcome but is not satisfactorily valid.

However, for ICU treatment extending past the normal postoperative observational period of 1 to 3 days, we found considerable differences in mortality and nursing-care need after 6 months and 1 year. Particularly, patients requiring CVVHDF or IVM had disastrous long-term results.

Data dealing with outcome parameters concerning patients obtaining CVVHDF- and IVM-dependent complications remain sparse, but the current investigation adds information that might be helpful in decision making serving this vulnerable patient cohort.

## Figures and Tables

**Table 1:** Baseline data, given as mean and standard deviation (±) or as odds ratio with confidence interval (CI). For all tests, statistical significance was assumed at *p* ≤ 0.05.

	Given	nICU	sICU	pICU	*p* = sICU/pICU
Number of patients	*N* = 402	66	275	61	*—*

Patients' age	Mean (SD)	79 (±8)	82 (±8)	82 (±9)	*0.970*
	95% CI	77–81	81–83	80–84	*—*

Gender	Male (%)	14 (21%)	71 (26%)	24 (39%)	*—*
	Female (%)	52 (79%)	204 (74%)	37 (61%)	*0.049*

BMI	Mean (SD)	16 (±33)	18 (±29)	19 (±28)	*0.999*

ASA score	Mean (SD)	2.7 (±0.7)	2.9 (±0.6)	3.1 (±0.6)	*0.107*

Prefracture Barthel index	95% CI	2.5–2.8	2.9–3.0	2.9–3.2	*—*
	Mean (SD)	89 (±21)	79 (±24)	72 (±28)	*0.300*

Prefracture nursing-care need	95% CI	83–94	76–82	65–80	*—*
	%	21 (32%)	144 (52%)	44 (72%)	0.042

Charlson score	Mean (SD)	1.7 (2.2)	2.3 (2.1)	3.6 (2.8)	<0.001
	95% CI	1.1–2.2	2.0–2.5	2.9–4.3	—

Time to operation/h	Mean (SD)	18.1 (1.4)	17.2 (0.8)	23.3 (2.3)	0.041
	95% CI	15.2–21	15.7–18.7	18.7–27.9	—

**Table 2:** Mortality and nursing care dependence of ICU length of stay. *∗*: survivors. For all tests statistical significance was assumed at *p* ≤ 0.05.

	nICU	sICU	pICU	*p* = sICU/pICU
In-hospital mortality	0 (0)	9 (3%)	16 (26%)	<0.001
6-month mortality^*∗*^	4 (6.1%)	48 (18%)	29 (48%)	<0.001
12-month mortality^*∗*^	9 (15.5%)	60 (26%)	33 (59%)	<0.001
6-month nursing-care need^*∗*^	22 (42%)	123 (67%)	23 (85%)	*0.073*
12-month nursing-care need^*∗*^	16 (43%)	101 (66%)	16 (84%)	*0.123*

**Table 3:** Multivariate analysis of independent risk factors for in-hospital mortality (IHM), 6-month mortality (6 MM), and 12-month mortality (12 MM). OR: odds ratio; CI: confidence interval; BMI: body mass index.

Variables Point of analysis	OR IHM	OR 6 MM	OR 12 MM	95% CI IHM	95% CI 6 MM	95% CI 12 MM	*p* value IHM	*p* value 6 MM	*p* value 12 MM
Age	0.980	1.031	1.032	0.919–1.004	0.990–1.073	0.994–1.072	0.529	0.145	0.096
Gender	1.080	0.582	0.653	0.330–3.534	0.304–1.115	0.394–1.223	0.899	0.103	0.184
Barthel index	1.000	0.997	0.992	0.979–1.021	0.984–1.009	0.980–1.004	0.983	0.594	0.173
ASA	3.085	2.147	2.160	1.059–8.985	1.190–3.873	1.241–3.758	**0.039**	**0.011**	**0.006**
Charlson index	1.048	1.160	1.196	0.847–1.296	1.013–1.328	1.050–1.362	0.666	**0.032**	**0.007**
BMI	0.993	1.001	1.003	0.978–1.007	0.991–1.012	0.993–1.013	0.327	0.830	0.557
Time until operation	1.001	1.002	1.000	0.969–1.003	0.982–1.023	0.980–1.020	0.976	0.826	0.988
ICU cohort	20.493	3.221	2.112	5.835–71.981	1.792–5.793	1.235–3.614	**0.000**	**0.000**	**0.006**
Constant	0.000	0.001	0.003	—	—	—	0.019	0.002	0.005

**Table 4:** Reasons for admission to ICU in pICU cohort.

Reasons for ICU treatment > 3 days	Number of patients
Cardiac arrhythmia	5
Cardiac ischemia	8
Cardiac failure	4
Renal failure	8
Cholezystitis/cholangitis	3
Respiratory failure	10
Infection	9
Bowel pathology with need for surgical intervention	6
Epilepsy	1
Operative revision of surgical complication	2
Hepatic failure	1
Postoperative anemia/impaired coagulation	4

**Table 5:** Mortality and nursing-care need after different assessments in pICU cohort. *∗*: survivors.

	Need for renal filtration	Need for invasive ventilatory management
Number of patients	*N* = 11	*N* = 25
In-hospital mortality	9 (82%)	17 (68%)
6-month mortality^*∗*^	9 (82%)	22 (88%)
12-month mortality^*∗*^	10 (91%)	22 (92%)
6-month nursing-care need^*∗*^	2 (100%)	3 (100%)
12-month nursing-care need^*∗*^	1 (100%)	2 (100%)
